# Do material efficiency improvements backfire?: Insights from an index decomposition analysis about the link between CO_2_ emissions and material use for Austria

**DOI:** 10.1111/jiec.13076

**Published:** 2020-10-14

**Authors:** Barbara Plank, Nina Eisenmenger, Anke Schaffartzik

**Affiliations:** ^1^ Institute of Social Ecology (SEC), Department of Economics and Social Sciences (WiSo) University of Natural Resources and Life Sciences (BOKU) Vienna Austria

**Keywords:** carbon emissions, decoupling, industrial ecology, material footprint, multi‐regional input–output (MRIO) model, supply chains

## Abstract

To keep global heating and other negative consequences of socioeconomic activities within manageable boundaries, industrialized countries must undergo substantial decarbonization, requiring the exploitation of synergies with other environmental endeavors. Improving resource efficiency—that is, reducing the resources required to generate a unit of economic output—is a prominent goal pursued across levels of scale. How does resource efficiency relate to decarbonization? Do economies decrease their emissions as they become more efficient? We examine this relationship for Austria from 2000 to 2015 by conducting an index decomposition analysis at the sectoral level by using consumption‐based indicators from the multi‐regional input–output model Exiobase. Our analysis shows that for Austria, the currently popular pursuit of material efficiency appears to run the risk of coinciding with higher emissions, suggesting that the opportunities to achieve both decarbonization and dematerialization are limited. The Austrian service sectors could contribute to a reduction of the CO_2_ footprint via material efficiency improvements, but strong economic growth foils this possibility coming to fruition. The Austrian economy would do well to either curb demand for goods and services driving global CO_2_ emissions or to produce imported goods and services domestically in an environmentally more benign manner.

## 1 INTRODUCTION

The climate crisis is among the most important environmental problems for society. CO_2_ emissions contributing substantially to this crisis occur at every step of supply and use of goods and services. To keep negative consequences of socioeconomic activities such as global heating within manageable boundaries, industrialized countries must undergo substantial decarbonization now. This in and of itself is a fundamental challenge to current modes of societal organization. Adding to the challenge is the fact that the climate crisis is part of a deep crisis of society‐nature relations (Hummel, Jahn, Keil, Liehr, & Stieß, 2017; Pichler, Schaffartzik, Haberl, & Görg, 2017). Decarbonization strategies are required that are compatible with curbing natural resources extraction and alleviating rather than aggravating the associated socioecological distribution conflicts (Martinez‐Alier, 2009; Scheidel, Temper, Demaria, & Martínez‐Alier, 2018). It has been proposed that by increasing resource efficiency, that is, by reducing the amount of resources required to generate each unit of economic output, it may be possible to simultaneously reduce greenhouse gas (GHG) emissions and material resource extraction (IRP, 2017). For proponents of economic growth as a non‐negotiable societal goal, resource efficiency promises the compatibility of this goal with much‐needed absolute decarbonization and dematerialization of economies (Hickel & Kallis, 2019; IRP, 2017) in the hopes of keeping extraction of natural resources and wastes and emissions within manageable limits (Rockström et al., 2009; Steffen et al., 2015). A departure from past trajectories and current trends should be achieved through material efficiency measures that increase infrastructure and product lifetimes, allow for intensified use, reduce energy and material requirements of production and use, and are conducive to reusing, remanufacturing, and recycling (Allwood, Ashby, Gutowski, & Worrell, 2011).

Efficiency indicators generally reflect developments with regard to one type of environmental pressure such as material extraction or GHG emissions. While overarching frameworks such as the UN Sustainable Development Goals (SDGs; UN, 2015) require that material efficiency improvements be achieved (SDGs 8 & 12) *whilst* curbing climate change (SDG 13), advocated by the UN Paris Agreement (UN, 2016), too little is known about possible synergies and trade‐offs between these (and other) targets (Nilsson, Griggs, & Visbeck, 2016). Attempts to mitigate emissions from materials processing often focus on supply‐side measures, such as improved energy efficiency in production processes, a shift toward cleaner energy sources, CO_2_ capture, and the use of alternative supply routes and of raw materials with lower embodied emissions (IEA, 2019). Demand‐side measures, that is reducing both intermediate and final demand for materials, can additionally contribute to mitigating emissions and can be achieved by material efficiency and sufficiency strategies (Worrell, Allwood, & Gutowski, 2016). Research on and development of demand‐side material efficiency measures has progressed markedly during the past decade (Zhang, Chen, & Ruth, 2018). Attempts were made to evaluate the potential contribution of material efficiency to meeting climate change mitigation targets and to attaining a global emission reduction of approximately 30% through efficiency‐induced reductions in demand for steel, cement, and aluminum (IEA, 2019). Several studies also found substantial emission reduction potentials of material efficiency measures to complement climate policies on the national level (e.g., Dente, Aoki‐Suzuki, Tanaka, & Hashimoto, 2018; Hernandez, Cooper‐Searle, Skelton, & Cullen, 2018; Kuramochi, 2016; Lin & Ouyang, 2014; Scott, Giesekam, Barrett, & Owen, 2018). Despite their potential effectiveness, material efficiency strategies have generally been overlooked in climate policies (Hernandez et al., 2018).

Material efficiency strategies affect not only energy demand and emissions during material processing, but also the operational energy use of the material products. Recent findings show systematic trade‐offs between material use in the production of buildings, vehicles, and appliances and energy use in their operation (reviewed by Hertwich et al., 2019). A systems perspective is therefore indispensable. In this article, we propose that the social metabolism concept offers an analytical frame conducive to the identification of possible synergies and trade‐offs between different sustainability goals and targets, specifically in light of the need for decarbonization and dematerialization of the global economy in a socially just manner. Social metabolism entails the material and energy inputs, transformation, and outputs required by societies for their reproduction (Haberl et al., 2019). Societies gain resource inputs through extraction (mainly via agriculture and forestry and mining) and imports and either accumulate these inputs as stocks or transform them into wastes and emissions through use, or into outputs to other economies as exports. Any resource input translates to a waste or emission output—the question is how rapidly this transformation occurs. Resource use and resource efficiency policies tend to address the input side of social metabolism, whereas climate policies tend to target emissions on the output side. But, of course, it is the carbon contained in societies’ inputs (e.g., in coal, oil, or fuel wood) that is (eventually) transformed into the carbon content of emissions (e.g., in CO_2_ or CO), so that emission reduction targets could be translated into the corresponding input reductions.

The composition and extent of current global social metabolism are already pushing or crossing planetary boundaries (Rockström et al., 2009; Steffen et al., 2015). Studies on future resource use project that global material demand could double by 2050 (IRP, 2019; Krausmann et al., 2018), which would require increasing amounts of energy for extraction, processing, and use of material goods. Some links between material demand and emissions are even expected to grow stronger, since the contribution of material processing to global GHG emissions increased from 15% in 1995 to 23% in 2015, with the largest contribution stemming from bulk material construction such as iron and steel, cement, lime and plaster, other minerals used for construction purposes, as well as plastics and rubber (Hertwich, 2019; UNEP, 2019). Both in order to achieve reduction targets for GHG emissions and to provide relief for the people and ecosystems threatened by socioecological distribution conflicts, the dematerialization of economies is necessary (Akenji, Bengtsson, Bleischwitz, Tukker, & Schandl, 2016). Climate change mitigation and resource use reduction must be treated in conjunction with one another.

In this paper, we consider synergies and trade‐offs between decarbonization and dematerialization for Austria as a case study, questioning the role that material efficiency can play in achieving these objectives. Austria ratified the Kyoto Protocol and published the first Federal Climate Strategy in 2002 (BMFLUW, 2002), accentuated with the Climate and Energy Strategy 2030 (BMNT, 2018) in 2018. The latter sets a new target to reduce Austrian GHG emissions by 36% between 2005 and 2030. Among the EU28 countries, Austria had the 11th highest per‐capita emissions in 2016 (Eurostat, 2019). Austrian territorial CO_2_ emissions grew by 7% between 1990 and 2015. Between 2005 and 2014, a change in trends was observed: Austrian CO_2_ emissions peaked in 2005 and then declined as a result of a reduction of fossil fuel use in thermal power plants, improvements in the thermal quality of buildings, and the reduction of fossil fuel use in heating systems (UBA, 2018). However, from 2014 onward, CO_2_ emissions were on the rise, again. So far, climate change policies did not succeed in changing carbon emissions toward a trajectory compatible with the 1.5°C target.

Resource efficiency policies are an even more recent development than climate policy. In the EU, the flagship initiative “A resource efficient Europe” (European Commission, 2011a) and the roadmap “Towards a resource efficient Europe” (European Commission, 2011b) were published in 2011 but without a specific target. More recently, resource efficiency targets were included in the UN SDGs (UN, 2015), requiring the reduction of resource use and the improvement of resource efficiency (measured as domestic material consumption or material footprint per GDP), but no numerical target was set here either.^1^ In the past two decades, Austrian domestic material consumption stagnated but did not decline, with non‐metallic minerals consumption decreasing and metals increasing (BMFLUW, 2015). In order to change trends toward decarbonization and dematerialization, additional effort is needed; an integrated policy program could contribute to the implementation of effective measures and to avoid a deadlock by investing into contradictory measures.

This study focuses on the links between Austria's material footprint and CO_2_ emissions. Austria is an example of an import‐dependent industrialized economy with around 80–90% of fossil fuels and metallic goods originating in foreign economies, making it crucial to consider upstream resource requirements along international supply and use chains to obtain a comprehensive picture of Austrian resource requirements. Windsperger et al. (2019), for instance, show that Austrian consumption‐based emissions occur primarily along the production chain of products for Austrian final consumption, of which imported goods account for the largest share (42% of total consumption‐based emissions). In our analysis, we capture upstream environmental pressures through consumption‐based CO_2_ and material footprint indicators (Wiedmann & Lenzen, 2018). Footprint indicators have been developed to reflect the direct and indirect provisioning or appropriation of resource extraction or emissions to or from the global economy (Afionis, Sakai, Scott, Barrett, & Gouldson, 2017). Additionally, we identify dynamics of and trade‐offs between particular sectors within the economy, which are more commonly addressed by policy than the national level is (Girod, 2016). At the national and the sectoral level, we identify synergies and trade‐offs between dematerialization and decarbonization. Does improved material efficiency help reduce the CO_2_ footprint? How do international outsourcing tendencies affect material flows and emissions?

With an index decomposition analysis (IDA) (Xu & Ang, 2013) of an alternative formulation of the Kaya equation (Waggoner & Ausubel, 2002), including material efficiency as a driver of the CO_2_ footprint, we investigate the relationships between the carbon and the material footprint and gross value added (GVA) for Austria's main economic sectors. Section 2 provides an overview of the method and data, Section 3 presents results for the national and the sectoral level, and Section 4 discusses implications for policy.

## 2 METHODS

In this study, we analyzed the changes in material footprint and CO_2_ footprint and derived efficiencies for Austria in total and for 60 economic sectors between 2000 and 2015. To identify underlying trade‐offs and synergies, we conducted an IDA at the sectoral level, using consumption‐based indicators derived from the multi‐regional input–output (MRIO) database Exiobase 3 (Stadler et al., 2018). The analyzed relationship is a modification of the well‐known Kaya identity, which identifies efficiency factors and economic growth as underlying drivers of emissions (Kaya & Yokobori, 1998; Waggoner & Ausubel, 2002). We integrated the material footprint into the decomposition equation for the Austrian CO_2_ footprint. Carbon and material footprints indicate the total amount of CO_2_ emissions or raw materials extracted (measured in metric tons) associated with final demand in a country, that is, private and government consumption, investments, and changes in inventories (Peters, Minx, Weber, & Edenhofer, 2011; Wiedmann et al., 2015). The footprints are calculated by using the environmentally extended MRIO model Exiobase (Stadler et al., 2018). We furthermore differentiated carbon and material footprints into two parts: either delivered by domestic or by foreign sectors to Austrian final demand. Based on these results, we will discuss the potential contribution of resource efficiency to emissions savings in different economic sectors and the potential of efficiency strategies to outweigh outsourcing tendencies.

The sector definitions applied in our analysis differ from the conventional territorial emission accounting framework, which is employed by the United Nations Framework Convention of Climate Change (UNFCCC, 2015). The sectoral analysis follows the 59 NACE (Nomenclature statistique des activités économiques dans la communauté Européenne, Statistical Classification of Economic Activities in the European Community) sectors (Statistik Austria, 2008), which, upon completion of the IDA, we aggregated to seven major economic activities, namely agriculture, mining, manufacturing, electricity and utilities, construction, transport, and services (the aggregation table is shown in the Supporting Information). Conventional GHG emission inventories, which are also known as production‐based accounts, record emissions released by agents (e.g., by industries, private households, governments) within the geographical borders of a nation (territory principle) or agents registered as residents of a nation (residence principle) (Usubiaga & Acosta‐Fernández, 2015). In our sectoral analysis, we focus on footprint indicators and by that on a consumption‐based perspective. Footprints at the sectoral level represent the upstream resource use or CO_2_ emissions of the goods and services delivered to final demand from these sectors. Consequently, footprints do not include the material extraction or emissions associated with goods and services produced for export. The sum of the sectoral footprints equals the national material or CO_2_ footprint.

A consumption‐based perspective on CO_2_ emissions and material extraction follows the logic that final consumption ultimately drives emissions and resource extraction, and thus allocates all emissions and resource extraction along international supply chains to final consumption and to the country in which this final consumption occurs (Hertwich & Peters, 2009; Wiedmann et al., 2015). In our analysis, we are concerned with the CO_2_ emissions directly occurring in the industrial production of consumer goods and services. These are the emissions that can be allocated to either production or the footprints of consumption, depending on the approach used. In industrial production, CO_2_ is directly emitted through combustion of fossil fuels (e.g., of coal in thermal power plants or blast furnaces) and through thermal decomposition in the production of cement or lime and iron reduction in steel‐making. CO_2_ emissions constitute by far the highest share of total GHG emissions (85% in Austria in 2015) and account for almost the entirety of GHG emissions in the sectors transport, energy, industry, and construction (UBA, 2019).

How the information provided by footprint indicators can be interpreted politically and economically is under debate (Afionis et al., 2017; Schaffartzik, Wiedenhofer, & Eisenmenger, 2015). Material and CO_2_ footprint indicators, as estimated based on environmentally extended MRIO models, are shaped both by domestic and foreign economic structures and by the geographic patterns of production and consumption (Plank, Eisenmenger, Schaffartzik, & Wiedenhofer, 2018). To be able to discuss entry points for policy interventions in a differentiated manner, we distinguished between the fraction of the footprint that is processed domestically, that is, material extraction and CO_2_ emissions that occur due to intermediate (and ultimately final) demand of the Austrian economy, and the fraction that is internationally appropriated via imports, that is, when material extraction and CO_2_ emissions are connected to products delivered to Austrian final demand by foreign sectors. The domestic fractions of the footprints also entail material extraction and CO_2_ emissions from foreign regions of upstream supply chains of Austrian sectors. They therefore represent the footprint shares referring to domestic economic value generation (in contrast to the imported fractions where economic value accrues abroad).

For the Austrian CO_2_ footprint (CF), our decomposition equations are based on the following functional form, distinguishing four explicit determinants:

$$\mathrm{CF}\;=\sum \left(A*S*\mathrm{ME}*\mathrm{EI}\right)\;=\sum_{i=1}^{n}\left(\mathrm{GDP}*\frac{\mathrm{GV}A_{i}}{\mathrm{GDP}}*\frac{MF_{i}}{\mathrm{GV}A_{i}}*\frac{CF_{i}}{MF_{i}}\right)$$

- *A*—economic growth effect: changes in Austrian GDP
- *S*—value added structure effect: changes in GVA structure (GVA*_i_/*GDP) per sector *i* 2
- ME—material footprint intensity effect: changes in material footprint per GVA of sector *i* (MF*_i_/*GVA*_i_*)^3^
- EI—emission‐to‐resource ratio effect: consumption‐based CO_2_ emissions per material footprint of sector I (CF*_i_/*MF*_i_*)

We used the following three equations to decompose the annual change in CF in total and on a sectoral level:

- Total CF decomposition:
  $$\Delta \mathrm{CF}\;=CF^{1}\;-\;CF^{0}=\Delta CF_{A}\;+\Delta CF_{S}+\Delta CF_{ME}+\Delta CF_{EI}$$
- Domestic CF decomposition:
  $$\Delta \mathrm{CF}dom\;=\mathrm{CF}dom^{1}\;-\;\mathrm{CF}dom^{0}=\Delta \mathrm{CF}dom_{A}\;+\Delta \mathrm{CF}dom_{S}+\Delta \mathrm{CF}dom_{ME}+\Delta \mathrm{CF}dom_{EI}$$
- Imported CF decomposition:
  $$\Delta \mathrm{CF}imp\;=\mathrm{CF}imp^{1}\;-\;\mathrm{CF}imp^{0}=\Delta \mathrm{CF}imp_{A}\;+\Delta \mathrm{CF}imp_{S}+\Delta \mathrm{CF}imp_{ME}+\Delta \mathrm{CF}imp_{EI}$$

IDA is a widely accepted tool to study the impacts of sectoral intensity changes and other economic structural changes on trends in emissions and energy use (Xu & Ang, 2013). Generally speaking, a decomposition analysis dissects the underlying factors determining the development of a certain endogenous variable (e.g., CO_2_ emissions or other pollutants) (Dietzenbacher & Los, 1998). It assumes that there is a functional dependency between the exogenous underlying factors and the endogenous variable, which can be decomposed into the changes between two points in time determined by each factor using differential calculus (Hoekstra & van den Bergh, 2002). Here, we studied the constituent factors that determine the development of the CO_2_ footprint (ΔCF) as the endogenous variable. The resulting factors can be interpreted as *ceteris paribus* effects, that is, how much the CF would have changed if only one specific underlying factor had changed as it actually did while all other factors remained constant. As the decomposition method, we chose logarithmic mean Divisia index (LMDI) I (for implementation of the method see, e.g., Ang, 2015).

Footprint indicators are calculated by using the environmentally extended MRIO model with the highest, consistent sector and product detail available: Exiobase (Stadler et al., 2018; Wood et al., 2018). Exiobase version 3.6 integrates 44 countries and 5 rest‐of‐the‐world regions and covers 163 industries for the years from 1990 to 2015. Monetary data on sectoral GVA in constant prices (2015 chained linked volumes) was taken from Statistik Austria (2019). Further information on the calculation of footprints and input–output modeling can be found in Miller and Blair (2009).

The specific methods (MRIO modeling, IDA) clearly have certain limitations, which potentially affect the results, due to dependency problems, issues related to sector and spatial aggregation and IO assumptions, which are elaborated in the literature (Ang, 2015; Miller & Blair, 2009; Wiedmann, Wilting, Lenzen, Lutter, & Palm, 2011; Wieland et al., 2019). Further detailed information on the methodology and equations for footprint and decomposition calculations can be found in Supporting Information S1.

## 3 RESULTS

Along production chains and in final consumption, high material use and high CO_2_ emissions do not necessarily coincide: materials are extracted in the primary sectors and distributed to further production and consumption while CO_2_ is emitted throughout production and consumption. In our case study, we are concerned with how these differences in material and CO_2_ flows relate not only to one another but also to the generation of economic value.

### 3.1 Austria in the global economy

For almost all metal and fossil fuel resources, Austria depends on imports. This is to be expected of a densely populated industrialized country in which extraction occurs mainly through agriculture and forestry and for construction minerals. The material footprint (MF) and the CO_2_ footprint (CF) are higher than domestic material consumption or territorial emissions; Austria draws on natural resources used in production processes abroad. In the year 2015, the Austrian MF of 216 Megatons (Mt, 1 Mt = 10^6^ tons) or 25 tons per capita (t/cap) was significantly higher than domestic material extraction (158 Mt and 18 t/cap). The CF amounted to 88 Mt (10 t/cap) while territorial CO_2_ emissions accounted for 65 Mt (8 t/cap). In the European context, Austria is among the countries of high resource requirements and inducing high emissions (rank 11 for both the MF and the CF) (UNE IRP, 2019; Wood, Moran, Rodrigues, & Stadler, 2019).

**FIGURE 1:**
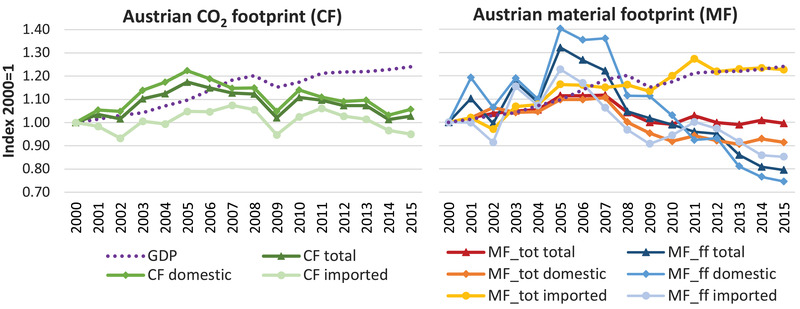
Development of Austrian total, domestic, and imported CO_2_ (CF) and material footprint (MF) and GDP from 2000 to 2015, indexed for 2000 = 1. MF_tot comprises all material categories, MF_ff only includes fossil fuels. Underlying data used to create this figure can be found in Supporting Information S2

Over the last two decades, Austria's MF and CF remained relatively stable (Figure 1). The Austrian CF grew between 2000 and 2005, followed by a slight and then drastic reduction leading up to and following the 2008 economic crisis. With the exception of 2015, the CF shows decoupling from GDP after 2010. The MF followed a similar general pattern but decoupling from GDP occurs after 2007. The MF covers diverse materials (biomass, fossil fuels, metallic and non‐metallic minerals) Of those, the fossil material footprint as a fraction of total MF is directly relevant for CO_2_ emissions, since most fossil fuels are combusted. The fossil MF strongly decreased after 2007, and by 2015, was 20% lower than it had been in 2000.

The overall trajectory of Austria's CF and MF is the result of differing patterns in the domestic and the imported fraction of these indicators (Figure 1). Austria's CF experienced different phases of growth and decline from 2000 to 2015. Overall, it was 2.4 Mt larger in 2015 than in 2000. The domestic fraction of the CF grew more pronouncedly than total CF, out‐performing reductions in the imported CF. In 2015, the domestic CF was higher, whereas the imported CF was lower than in 2000. For the MF, the reverse can be observed: The domestic MF decreased while the imported MF grew. For the fossil fuel fraction of the MF, the domestic share is primarily responsible for the strong decrease after 2007. While Austrian domestic production seems to require less fossil fuel extraction, the same does not apply for the Austrian imports.

**FIGURE 2:**
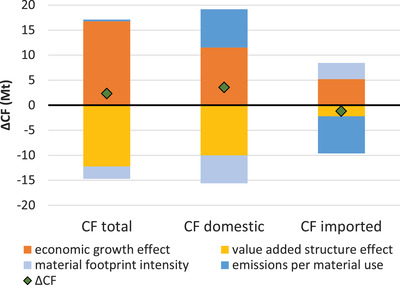
Decomposition of the Austrian CO_2_ footprint (CF) change from 2000 to 2015, for the total CF and divided into the domestic and the imported fraction of the CF. Underlying data used to create this figure can be found in Supporting Information S2

In light of the necessary decarbonization of the Austrian economy, we use an IDA to circle in on those factors that drive the CF, uncovering the role—if any—that the MF plays therein. CF growth was mainly driven by economic growth (operationalized as growth in GDP) (Figure 2). If all else had remained equal, economic growth alone would have caused the Austrian CF to increase by over 15 Mt (or 24 %). However, changes in the value added structure of the Austrian economy would have caused an almost equal decrease in the CF. Whether this means that Austria's economic growth increasingly stems from sectors that are less carbon intensive is examined in Section 3.2 of this article. Economic growth and value added structure act as opposing forces in both the domestic and the imported fraction of the CF. The CF/MF would, *ceteris paribus*, cause the domestic CF to increase substantially (by 7.7 Mt) and the imported CF to decrease by almost the same amount (7.4 Mt). In both cases, the material footprint per value added acts as an opposing force to the CF/MF, reducing the domestic CF but increasing the imported CF. These results indicate that Austrian domestic production has become more material efficient but the carbon intensity of the materials used increased, while exactly the opposite is true for Austrian imports. For a further investigation we will leave the national level and look at economic sectors and their interlinkages.

### 3.2 The sectoral perspective

Differentiating the Austrian economy by seven major sector aggregates—agriculture, mining, manufacturing, electricity and utilities, construction, transport, and services—we find that total CO_2_ and material footprints are overarchingly influenced by manufacturing and service sectors. Manufacturing sectors account for approximately 30–40% of the total CF and MF and service sectors for more than 20%. Construction and mining are equally important for the MF with slightly less than 15% each, while the electricity sector ranks third for the CF with 10%. The construction sector accounts for 8% and the transport sector for 7% of the CF. In addition to the emissions from the production chain, the CF also encompasses CO_2_ emissions that are emitted directly by households and the government (e.g., mobility, heating; all together 20% of CF). Those emissions remained rather stable over the observed time period and have been included in the domestic CF in Figures 1 and 2. However, these direct emissions are not included in the following analysis where we focus on the production of goods and services delivering to Austrian final demand.

**FIGURE 3:**
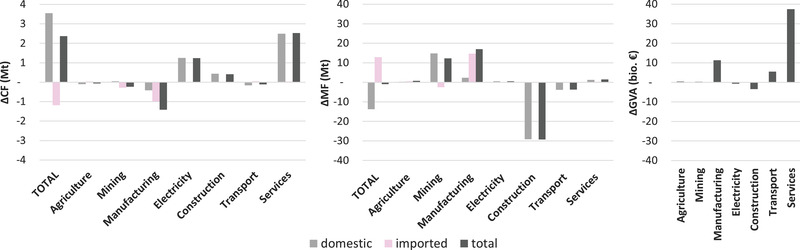
Changes of total, domestic, and imported CO_2_ (CF) and material footprint (MF) and gross value added (GVA) for main sector aggregates in Austria from 2000 to 2015. Underlying data used to create this figure can be found in Supporting Information S2

The *growth* in Austria's overall CF can be ascribed to three sectors: services (60%), electricity (30%), and construction (10%) (Figure 3). These are also the sectors in which the growth of Austria's domestic CF is rooted. The decrease in the imported CF, in contrast, mainly stems from manufacturing. The MF is primarily influenced by a strong increase in the imported fraction of manufacturing as well as the domestic fraction of mining; the domestic fraction of construction counterbalances this, so that total MF slightly decreases. Since these sectors also dominate the overall economic growth of the Austrian economy, their behavior in terms of emissions and material use potentially has far‐reaching impacts: services accounted for over 50% of GDP in 2015, while also showing the strongest economic growth, and transport and manufacturing for about 20% each; in total, these sectors account for more than 90% of Austrian GDP. Most of the sector groups represent an aggregate of a number of sectors; the construction sector represents only one NACE sector, but still has a share of 6% of total GDP in 2015, although its GVA slightly decreased from 2000 to 2015.

**FIGURE 4:**
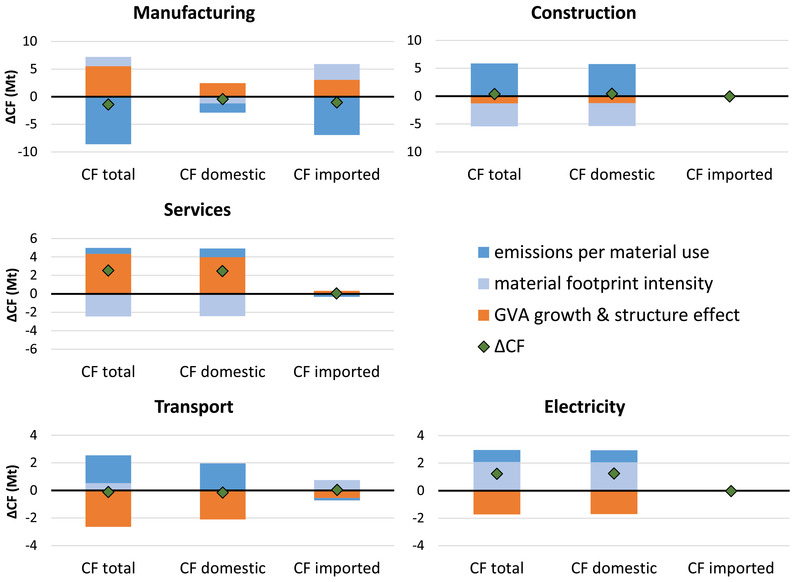
Decomposition of the total, domestic, and imported CO_2_ footprint (CF) change for five major economic sectors/sector aggregates in Austria from 2000 to 2015. Underlying data used to create this figure can be found in Supporting Information S2

At the aggregate level, we observed from the decomposition analysis that economic growth and value added structure tend to act as opposing forces as do CF/MF and MF intensity, although for the latter in opposite directions for imported and domestic CF (Figure 2). At the level of the sectors, we find that, among the dominantly influential sectors presented in Figure 3, increases in the CF that are caused by economic growth outstrip reductions due to value added structure effects for services and manufacturing sectors (Figure 4). Reductions due to the structure effect are greater than increases due to economic growth for transport sectors. Construction and electricity sectors decline in GVA, contributing, together with structure effects, to a decrease in CF.

CF/MF and MF intensity tend to act in opposite directions at the sectoral level as well, although within the sectors the difference between imported and domestic CF is not clear cut, again providing empirical support for our proposition that the aggregate trends in CF must be understood in terms of the different composition (especially due to non‐competitive imports) of domestic production and imports. CF/MF would, *ceteris paribus*, cause significant reductions in the CF of manufacturing while the same variable would cause the CF of all other sectors presented here to increase (although often to a lesser degree, note the different scales of the *y*‐axes in Figure 4). For manufacturing, not even the (additional) growth that MF intensity would cause offsets these potential reductions, so that the CF associated with manufacturing overall decreases. In services, overall CF growth relates especially to the growth of the domestic fraction of the CF where economic growth outstrips the structure effect and cannot be offset by the reduction that MF intensity could cause. For the Austrian aggregate, we noted that economic growth might especially have occurred in the sectors that are less CO_2_ intensive (indicated by the negative structure effect in Figure 2). Contrary to what may be a popular belief, these are *not* the service activities.

The perspective on aggregate sectors taken in the analysis of the data in Figure 4 suggests that economic growth and the changes in value added structure are linked to increased environmental impact in some groups of sectors but not in others. MF intensity may or may not play a role in curbing emissions and CF/MF can cause the CF to increase (e.g., for construction) or decrease (e.g., for manufacturing). Like many mature capitalist economies, Austria has been experiencing a shift from the secondary sectors (such as manufacturing) to the tertiary (service) sectors. As noted above, the growth in the services between 2000 and 2015 has been accompanied by a rise in the CF, mostly through emissions occurring within Austria. Manufacturing, which continues to experience economic growth but not to the extent that is seen of services (Figure 3), has experienced the most notable reductions in its CF, reflecting developments outside of Austria more strongly than domestic ones.

**FIGURE 5:**
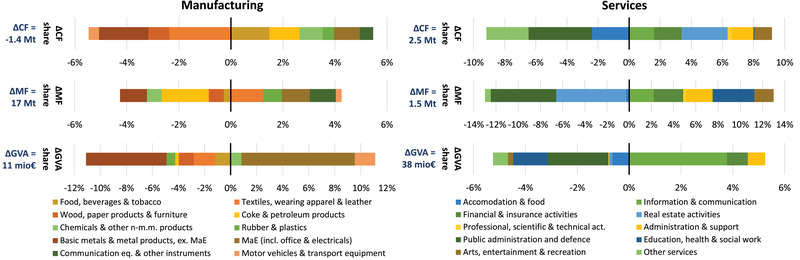
Changes in the sector mix of the manufacturing and service sector aggregate for the CO_2_ footprint (CF), the material footprint (MF), and gross value added (GVA) from 2000 to 2015. MaE, machinery and equipment. Underlying data used to create this figure can be found in Supporting Information S2

In order to delve more deeply into the analysis of what is growing and how in services and manufacturing, we consider changes between 2000 and 2015 in the share of more detailed groups of sectors in the CF, the MF, and value added of manufacturing and services (Figure 5). We find sectors for which decreased shares in value added (bar at the bottom in Figure 5) corresponded to decreased shares in the CF and MF (top two bars) or, conversely, increased share in value added corresponded to higher shares in CF and MF. Among the manufacturing sectors, the former can be observed for basic metals and metal products as well as wood and paper products, the latter for machinery and equipment (MaE). But we also find evidence for less clear‐cut relationships: textiles’ share in VA decreases (as does share in the CF) while share in MF increases; coke and petroleum products see a slight decline in value added share, a strong decline in MF share and an increase in CF share.

Among the services, the correspondence between growth in all shares is given for information and communication technologies (ICT), for financial and insurance activities, and for administrative and support services. From the small‐scale devices to large‐scale infrastructures commanded by ICT to the administrative and support services which may be labor intensive but also cover more material‐ and energy‐intensive rental services, it becomes apparent here that the services are not in general immaterial. We also see the reduced share of public administration and defense and—to a less pronounced extent—for accommodation and food coinciding with decreased shares in MF and CF. Examples of varied changes in shares are real estate activities as well as education, health, and social work.

## 4 DISCUSSION

At the most aggregate level, Austria's global claim to material resources (as represented by the material footprint) behaves quite differently from its contribution to the climate crisis (represented by the CO_2_ footprint). While Austria's MF has decreased slightly, the international fraction of MF has increased and been counterbalanced by the decreasing domestic fraction. The CF reflects increasing emissions in domestic production, while the emissions associated with imported goods and services decreased slightly. At the sectoral level, we find many instances of sectors that either make a high contribution to the MF *or* to the CF. But we only see one sector group where both MF and CF decrease: the transport sector, although with marginal decreases in CF (−2%). We also identify one sector aggregate for which both indicators increase: services. There, the MF increases slightly (+3%) and growth in the CF is more pronounced (+14%). This suggests that opportunities to achieve decarbonization and dematerialization with only one measure are limited at best. In fact, the possibility must be entertained that interventions to reduce CO_2_ emissions could increase material extraction if strict measures to avoid such trade‐offs are not developed and enforced.

The currently popular pursuit of (material) resource efficiency appears to run the risk of increasing emissions. The service orientation of the economy is frequently advertised as environmentally beneficial and in particular as a measure to curb material resource requirements (Canas, Ferrão, & Conceição, 2003). This, of course, depends on what the services are based on: Banks’ funds may rely heavily on primary and secondary sector activities, investments affect material extraction and emissions, insurances are often related to material activities, etc. Despite the functional dependence of many services on decidedly material (or not immaterial) sectors or products (e.g., information and communication requires electricity, hardware, and high turnover, material‐ and energy‐intensive appliances in final consumption) (Carmona, Whiting, Carrasco, Sousa, & Domingos, 2017), our analysis suggests that here, material efficiency improvements could indeed cause a reduction of the CF. It is precisely the immense economic growth of these sectors, however, coupled with the tendency of the emissions incurred per unit of MF to cause a rising CF, that links them to the rise of the Austrian CF. As is also the case for electricity, the contribution of the service sectors to the rising Austrian CF stems notably from domestic production (rather than imports). Service sectors also remain material and CO_2_ intensive due to their intense expansion and usage of societal material stocks (Pauliuk & Müller, 2014). Societal stocks represent substantial material flows and also their use (buildings, infrastructures) is responsible for high CO_2_ emissions (Müller et al., 2013). More empirical data on and analysis of physical stocks is currently at the center of international research (Lanau et al., 2019) and will contribute interesting new insights on cross‐cutting issues (Haberl, Wiedenhofer, Erb, Görg, & Krausmann, 2017). To date we can say that to compensate for sustained economic growth, resource efficiency measures have to focus on the stabilization or even reduction of societal material stocks and of their material and energy requirements (Krausmann et al., 2017).

Our analysis suggests that it may be advisable to develop specific approaches to target emissions and material requirements according to sectors and the goods and services they generate. This refers not so much to the technological (and technical) differences in opportunities for environmental protection but to the structural meta‐level distinctions that can be made with regard to Austrian production as compared to production elsewhere and to the relationships between economic growth, efficiencies, and intensities at the sectoral level. In the scientific literature, the argument prevails that net‐importing countries outsource environmental pressures (Simas, Pauliuk, Wood, Hertwich, & Stadler, 2017; Wiedmann & Lenzen, 2018). We find that it may not be import dependence as such but which specific goods and services an economy is import dependent for that relates closely to where environmental pressures occur. In the Austrian case, a large share of manufactured products, alongside their MF and CF, are imported. On the other hand, the environmental burden associated with the production of most of the other goods and services mostly puts pressure on the Austrian environment. This could mean that the Austrian economy would do well to seek ways to either curb final demand for or to domestically produce in an environmentally more benign manner those specific imports currently associated with driving fossil fuel consumption and CO_2_ emissions globally.

In light of continuous insufficiency of measures to address the climate crisis and reduce resource use, many questions regarding the effectiveness of policies on material efficiency remain. Demand‐side material efficiency contributions, along entire supply and use chains, to climate change mitigation could be achieved through a number of policies, including carbon taxation for bulk materials (Neuhoff et al., 2016), eco‐design laws (European Parliament, 2009), or circular economy strategies (European Commission, 2015). Domestic policies can influence environmental and social impacts of imports directly by trade mechanisms (via border trade adjustments), or indirectly by reducing environmental or social impacts abroad (e.g., linking emissions trading schemes or augmenting worldwide technology transfer), as well as implementing domestic actions focused on consumption (e.g., sustainable procurement measures) (Afionis et al., 2017). However, as also deduced from this analysis, not all policies aiming at resource efficiency or circular economy automatically co‐benefit climate change mitigation. It is crucial to gain a better understanding of the coupling of sectors and cascading of materials, including implications to material quality resulting from increased reuse and recycling (Hertwich et al., 2019). It is clear, however, that the underlying question is not one of sustaining growth by innovative means but of favoring policies and other measures now that are conducive to socioecological transformations. For example, not only do automobiles need to become less resource and emission intensive in their production and use, but they must be used within new systems of transport and corresponding changes to individual automobile ownership (Cherry, Scott, Barrett, & Pidgeon, 2018). Improved functional efficiency will always have the potential to backfire if it is not integrated into overarching processes of dematerialization and decarbonization in the context of socioecological transformation.

## CONFLICT OF INTEREST

The authors declare no conflict of interest.

## Supporting information


**Supporting Information S1**: This supporting information provides a summary of the methodological approach of the study and a concordance table for the sector classifications from Exiobase 3.6, the ÖNACE classification, and sector aggregates chosen for the article.

**Supporting Information S2**: This supporting information provides the results of our analysis shown in each figure in the main text of our article.

## Footnotes

1 Interestingly, the only consumption‐based environmental indicator that has been considered in the UN SDGs is not the carbon but the material footprint. Empirical analyses of material footprints and their interdependencies with other environmental pressures are therefore even more indispensable (Eisenmenger et al., 2020).

2 In our analysis, we show the value added structure effect for the total economy, but not at the sectoral level. The diversity within each sector varies widely. The value added structure effect is inconclusive at the sectoral level because it is hardly comparable between sectors. However, it is still included in a combined economic growth and structure effect.

3 In our globalized world, production and consumption of national economic sectors could be largely fragmented via international trade and economic value often accrues elsewhere than where final consumption occurs. From a consumption‐based perspective, the conceptually adequate economic indicator would therefore be final demand, and not gross value added (GVA) (Wood & Lenzen, 2009). Due to the unavailability of sectoral final demand in constant prices, we had to use GVA as the corresponding factor in our decomposition analysis. This conceptual inconsistence could bias our results for sectors where dynamics of final demand differ strongly from GVA dynamics. However, we found annual changes in sectoral final demand and GVA to be very much alike at our chosen level of sector aggregation and therefore expect our decomposition of changes over time to be rather robust to this bias.
