# Supplementary material for: Do material efficiency improvements backfire?: Insights from an index decomposition analysis about the link between CO2 emissions and material use for Austria
Source: J Ind Ecol. 2020 Oct 14;25(2):511–22. doi: 10.1111/jiec.13076 (PMC8247022; doi:10.1111/jiec.13076)
Supplement: Supplementary file 1 — Supporting Information S1: This supporting information provides a summary of the methodological approach of the study and a concordance table for the sector classifications from Exiobase 3.6, the ÖNACE classification, and sector aggregates chosen for the article. [file 44498_2021_2502019_MOESM1_ESM.pdf]

SUPPORTING INFORMATION FOR:

Plank, B., N. Eisenmenger, and A. Schaffartzik. 2020 Do material efficiency improvements backfire? Insights from an index decomposition analysis about the link between CO_2 emissions and material use for Austria. *Journal of Industrial Ecology.*

Summary

This supporting information provides a summary of the methodological approach of the study and a concordance table for the sector classifications from Exiobase 3.6, the ÖNACE classification, and sector aggregates chosen for the article.

1. Methodological approach

We analyzed changes in material and CO_2 footprints and derived efficiencies using the index decomposition method LMDI I. Index decomposition analysis (IDA) is a tool to study the impacts of sectoral intensity changes and other economic structural changes on trends in e.g. emissions and energy use in industries (Ang, 2004; Ang et al., 2009, 2010) and is widely accepted by policy-makers. A decomposition analysis dissects the underlying factors determining the development of a certain endogenous variable (e.g. CO_2 emissions or other pollutants) (Dietzenbacher & Los, 1998). It assumes that there is a functional dependency between the exogenous underlying factors and the endogenous variable, which can be decomposed into the changes between two points in time determined by each factor using differential calculus (Hoekstra & van den Bergh, 2002).

We started from the basic equation of environmentally extended input-output analysis (EEIOA) to calculate Austria's material (MF) and CO_2 footprint (CF). For the derivation of the equation and more information on EEIOA see, e.g., Miller and Blair (2009).

$$Footprint = f(I - A)^{-1}y = fLy \qquad \text{(S1)}$$

with the elements of *f* representing the materials or CO_2 necessary for one Euro production of sector j in each country, *L* representing the production of sector *j* in each country that is necessary for one Euro of consumption of sector *i* in a country (Leontief inverse) and *y* representing the Austrian final demand (i.e. private and government consumption, investments and changes in inventories). We furthermore distinguished between the part of the footprint that is processed domestically, i.e., when materials and CO_2 occur due to requirements of Austrian production sectors (that then ultimately deliver to Austrian final demand), and the part that is internationally appropriated via imports, i.e., when material extraction and CO_2 emissions are connected to products delivered to Austrian final demand by foreign production sectors. A schematic representation of the calculation of the footprints and the distinction between domestic and imported footprints is given in figure S1-1.

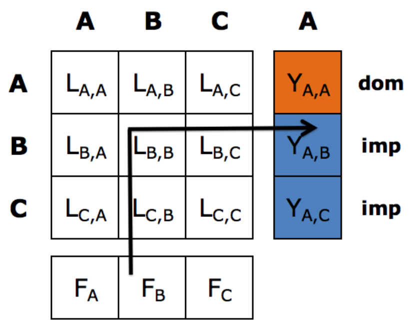

Figure S1-1. Scheme of the calculation of the domestic and the imported footprint in a multi-regional input-output model. Country A represents Austria in our study; orange indicates the domestic (dom), blue the imported (imp) fraction of the Austrian footprint.

Our decomposition equations are based on the following underlying functional form, distinguishing four explicit determinants:

$$CF = A * S * ME * EI = \sum_{i=1}^{n} GDP * \frac{GVA_i}{GDP} * \frac{MF_i}{GVA_i} * \frac{CF_i}{MF_i} \quad \text{(S2)}$$

- A – economic growth effect: changes in Austrian GDP
- S – value added structure effect: changes in gross value added structure (GVA_i/GDP) per sector i
- ME – material footprint intensity effect: changes in material footprint per value added of sector i (MF_i/GVA_i)
- EI – emission-to-resource ratio effect: consumption-based CO_2 emissions per material use of sector i (CF_i/MF_i)

In comparison to other IDA methods, the logarithmic mean Divisia index method (LMDI) is often preferred as it is easy to use and highly adaptive to different study designs (Ang, 2004). The basic formula for LMDI I in the additive form for the kth factor x for the aggregate variable V is:

$$\Delta V_{x_k} = \sum_i L\left(V_i^T, V_i^0\right) \times \ln\left(\frac{x_{k,i}^T}{x_{k,i}^0}\right) \quad \text{(S3)}$$

Where function $L(a,b)$ is the logarithmic average of two positive numbers a and b ($a \neq b$) given by

$$L(a,b) = \frac{a-b}{\ln a - \ln b} \tag{S4}$$

When we apply the LMDI additive decomposition to the four-factor decomposition we defined earlier, we arrive at following decomposition equations:

$$\Delta CF = CF^1 - CF^0 = \Delta CF_A + \Delta CF_S + \Delta CF_{ME} + \Delta CF_{EI} \tag{S5}$$

$$\Delta CF_A = L(CF^1, CF^0)\ln\left(\frac{A^1}{A^0}\right) \tag{S6}$$

$$\Delta CF_S = L(CF^1, CF^0)\ln\left(\frac{S^1}{S^0}\right) \tag{S7}$$

$$\Delta CF_{ME} = L(CF^1, CF^0)\ln\left(\frac{ME^1}{ME^0}\right) \tag{S8}$$

$$\Delta CF_{EI} = L(CF^1, CF^0)\ln\left(\frac{EI^1}{EI^0}\right) \tag{S9}$$

In addition to the IDA for the total Austrian footprint, we decomposed the domestic and the imported fraction of the material and the CO_2 footprint separately. The respective LMDI I equations have been changed to only include the domestic or the imported fractions of CO_2 as well as material footprint, resulting in two decompositions, which were as well calculated on the economy and on the sector level:

$$\begin{aligned}\Delta CFdom &= CFdom^1 - CFdom^0 \\ &= \Delta CFdom_A + \Delta CFdom_S + \Delta CFdom_{ME} + \Delta CFdom_{EI}\end{aligned} \tag{S10}$$

$$\begin{aligned}\Delta CFimp &= CFimp^1 - CFimp^0 \\ &= \Delta CFimp_A + \Delta CFimp_S + \Delta CFimp_{ME} + \Delta CFimp_{EI}\end{aligned} \tag{S11}$$

2. References

Ang, B. W. (2004). Decomposition analysis for policymaking in energy: Which is the preferred method? *Energy Policy*, *32*(9), 1131–1139. https://doi.org/10.1016/S0301-4215(03)00076-4

Ang, B. W., Huang, H. C., & Mu, A. R. (2009). Properties and linkages of some index decomposition analysis methods. *Energy Policy*, *37*(11), 4624–4632. https://doi.org/10.1016/j.enpol.2009.06.017

Ang, B. W., Mu, A. R., & Zhou, P. (2010). Accounting frameworks for tracking energy efficiency trends. *Energy Economics*, *32*(5), 1209–1219. https://doi.org/10.1016/j.eneco.2010.03.011

Dietzenbacher, E., & Los, B. (1998). Structural Decomposition Techniques: Sense and Sensitivity. *Economic Systems Research*, *10*(4), 307–324. https://doi.org/10.1080/09535319800000023

Hoekstra, R., & van den Bergh, J. (2002). Structural Decomposition Analysis of Physical Flows in the Economy. *Environmental and Resource Economics*, *23*(3), 357–378. https://doi.org/10.1023/A:1021234216845

Miller, R. E., & Blair, P. D. (2009). *Input-Output Analysis: Foundations and Extensions*. Cambridge University Press.

3. Table S1-1. Sector concordance table for the different classification systems used

Exiobase Industry Classification		ÖNACE Classification	Sector aggregates
i01.a	Cultivation of paddy rice	Agriculture, hunting and related service activities	Agriculture
i01.b	Cultivation of wheat	Agriculture, hunting and related service activities	Agriculture
i01.c	Cultivation of cereal grains nec	Agriculture, hunting and related service activities	Agriculture
i01.d	Cultivation of vegetables, fruit, nuts	Agriculture, hunting and related service activities	Agriculture
i01.e	Cultivation of oil seeds	Agriculture, hunting and related service activities	Agriculture
i01.f	Cultivation of sugar cane, sugar beet	Agriculture, hunting and related service activities	Agriculture
i01.g	Cultivation of plant-based fibers	Agriculture, hunting and related service activities	Agriculture
i01.h	Cultivation of crops nec	Agriculture, hunting and related service activities	Agriculture
i01.i	Cattle farming	Agriculture, hunting and related service activities	Agriculture
i01.j	Pigs farming	Agriculture, hunting and related service activities	Agriculture
i01.k	Poultry farming	Agriculture, hunting and related service activities	Agriculture
i01.l	Meat animals nec	Agriculture, hunting and related service activities	Agriculture
i01.m	Animal products nec	Agriculture, hunting and related service activities	Agriculture
i01.n	Raw milk	Agriculture, hunting and related service activities	Agriculture
i01.o	Wool, silk-worm cocoons	Agriculture, hunting and related service activities	Agriculture
i01.w.1	Manure treatment (conventional), storage and land application	Agriculture, hunting and related service activities	Agriculture
i01.w.2	Manure treatment (biogas), storage and land application	Agriculture, hunting and related service activities	Agriculture
i02	Forestry, logging and related service activities	Forestry, logging and related service activities	Agriculture
i05	Fishing, operating of fish hatcheries and fish farms; service activities incidental to fishing	Fishing, operating of fish hatcheries and fish farms; service activities incidental to fishing	Agriculture

i10	Mining of coal and lignite; extraction of peat	Mining of coal and lignite; extraction of peat	Mining
i11.a	Extraction of crude petroleum and services related to crude oil extraction, excluding surveying	Extraction of crude petroleum and natural gas; service activities incidental to oil and gas extraction excluding surveying	Mining
i11.b	Extraction of natural gas and services related to natural gas extraction, excluding surveying	Extraction of crude petroleum and natural gas; service activities incidental to oil and gas extraction excluding surveying	Mining
i11.c	Extraction, liquefaction, and regasification of other petroleum and gaseous materials	Extraction of crude petroleum and natural gas; service activities incidental to oil and gas extraction excluding surveying	Mining
i12	Mining of uranium and thorium ores	Mining of uranium and thorium ores	Mining
i13.1	Mining of iron ores	Mining of metal ores	Mining
i13.20.11	Mining of copper ores and concentrates	Mining of metal ores	Mining
i13.20.12	Mining of nickel ores and concentrates	Mining of metal ores	Mining
i13.20.13	Mining of aluminium ores and concentrates	Mining of metal ores	Mining
i13.20.14	Mining of precious metal ores and concentrates	Mining of metal ores	Mining
i13.20.15	Mining of lead, zinc and tin ores and concentrates	Mining of metal ores	Mining
i13.20.16	Mining of other non-ferrous metal ores and concentrates	Mining of metal ores	Mining
i14.1	Quarrying of stone	Other mining and quarrying	Mining
i14.2	Quarrying of sand and clay	Other mining and quarrying	Mining
i14.3	Mining of chemical and fertilizer minerals, production of salt, other mining and quarrying n.e.c.	Other mining and quarrying	Mining
i15.a	Processing of meat cattle	Manufacture of food products and beverages	Manufacturing
i15.b	Processing of meat pigs	Manufacture of food products and beverages	Manufacturing
i15.c	Processing of meat poultry	Manufacture of food products and beverages	Manufacturing
i15.d	Production of meat products nec	Manufacture of food products and beverages	Manufacturing
i15.e	Processing vegetable oils and fats	Manufacture of food products and beverages	Manufacturing

i15.f	Processing of dairy products	Manufacture of food products and beverages	Manufacturing
i15.g	Processed rice	Manufacture of food products and beverages	Manufacturing
i15.h	Sugar refining	Manufacture of food products and beverages	Manufacturing
i15.i	Processing of Food products nec	Manufacture of food products and beverages	Manufacturing
i15.j	Manufacture of beverages	Manufacture of food products and beverages	Manufacturing
i15.k	Manufacture of fish products	Manufacture of food products and beverages	Manufacturing
i16	Manufacture of tobacco products	Manufacture of tobacco products	Manufacturing
i17	Manufacture of textiles	Manufacture of textiles	Manufacturing
i18	Manufacture of wearing apparel; dressing and dyeing of fur	Manufacture of wearing apparel; dressing and dyeing of fur	Manufacturing
i19	Tanning and dressing of leather; manufacture of luggage, handbags, saddlery, harness and footwear	Tanning and dressing of leather; manufacture of luggage, handbags, saddlery, harness and footwear	Manufacturing
i20	Manufacture of wood and of products of wood and cork, except furniture; manufacture of articles of straw and plaiting materials	Manufacture of wood and of products of wood and cork, except furniture; manufacture of articles of straw and plaiting materials	Manufacturing
i20.w	Re-processing of secondary wood material into new wood material	Manufacture of wood and of products of wood and cork, except furniture; manufacture of articles of straw and plaiting materials	Manufacturing
i21.1	Pulp	Manufacture of pulp, paper and paper products	Manufacturing
i21.w.1	Re-processing of secondary paper into new pulp	Manufacture of pulp, paper and paper products	Manufacturing
i21.2	Paper	Manufacture of pulp, paper and paper products	Manufacturing
i22	Publishing, printing and reproduction of recorded media	Publishing, printing and reproduction of recorded media	Manufacturing
i23.1	Manufacture of coke oven products	Manufacture of coke, refined petroleum products and nuclear fuels	Manufacturing
i23.2	Petroleum Refinery	Manufacture of coke, refined petroleum products and nuclear fuels	Manufacturing

i23.3	Processing of nuclear fuel	Manufacture of coke, refined petroleum products and nuclear fuels	Manufacturing
i24.a	Plastics, basic	Manufacture of chemicals and chemical products	Manufacturing
i24.a.w	Re-processing of secondary plastic into new plastic	Manufacture of chemicals and chemical products	Manufacturing
i24.b	N-fertiliser	Manufacture of chemicals and chemical products	Manufacturing
i24.c	P- and other fertiliser	Manufacture of chemicals and chemical products	Manufacturing
i24.d	Chemicals nec	Manufacture of chemicals and chemical products	Manufacturing
i25	Manufacture of rubber and plastic products	Manufacture of rubber and plastic products	Manufacturing
i26.a	Manufacture of glass and glass products	Manufacture of other non-metallic mineral products	Manufacturing
i26.a.w	Re-processing of secondary glass into new glass	Manufacture of other non-metallic mineral products	Manufacturing
i26.b	Manufacture of ceramic goods	Manufacture of other non-metallic mineral products	Manufacturing
i26.c	Manufacture of bricks, tiles and construction products, in baked clay	Manufacture of other non-metallic mineral products	Manufacturing
i26.d	Manufacture of cement, lime and plaster	Manufacture of other non-metallic mineral products	Manufacturing
i26.d.w	Re-processing of ash into clinker	Manufacture of other non-metallic mineral products	Manufacturing
i26.e	Manufacture of other non-metallic mineral products n.e.c.	Manufacture of other non-metallic mineral products	Manufacturing
i27.a	Manufacture of basic iron and steel and of ferro-alloys and first products thereof	Manufacture of basic metals	Manufacturing
i27.a.w	Re-processing of secondary steel into new steel	Manufacture of basic metals	Manufacturing
i27.41	Precious metals production	Manufacture of basic metals	Manufacturing
i27.41.w	Re-processing of secondary preciuos metals into new preciuos metals	Manufacture of basic metals	Manufacturing
i27.42	Aluminium production	Manufacture of basic metals	Manufacturing
i27.42.w	Re-processing of secondary aluminium into new aluminium	Manufacture of basic metals	Manufacturing
i27.43	Lead, zinc and tin production	Manufacture of basic metals	Manufacturing
i27.43.w	Re-processing of secondary lead into new lead	Manufacture of basic metals	Manufacturing

i27.44	Copper production	Manufacture of basic metals	Manufacturing
i27.44.w	Re-processing of secondary copper into new copper	Manufacture of basic metals	Manufacturing
i27.45	Other non-ferrous metal production	Manufacture of basic metals	Manufacturing
i27.45.w	Re-processing of secondary other non-ferrous metals into new other non-ferrous metals	Manufacture of basic metals	Manufacturing
i27.5	Casting of metals	Manufacture of basic metals	Manufacturing
i28	Manufacture of fabricated metal products, except machinery and equipment	Manufacture of fabricated metal products, except machinery and equipment	Manufacturing
i29	Manufacture of machinery and equipment n.e.c.	Manufacture of machinery and equipment n.e.c.	Manufacturing
i30	Manufacture of office machinery and computers	Manufacture of office machinery and computers	Manufacturing
i31	Manufacture of electrical machinery and apparatus n.e.c.	Manufacture of electrical machinery and apparatus n.e.c.	Manufacturing
i32	Manufacture of radio, television and communication equipment and apparatus	Manufacture of radio, television and communication equipment and apparatus	Manufacturing
i33	Manufacture of medical, precision and optical instruments, watches and clocks	Manufacture of medical, precision and optical instruments, watches and clocks	Manufacturing
i34	Manufacture of motor vehicles, trailers and semi-trailers	Manufacture of motor vehicles, trailers and semi-trailers	Manufacturing
i35	Manufacture of other transport equipment	Manufacture of other transport equipment	Manufacturing
i36	Manufacture of furniture; manufacturing n.e.c.	Manufacture of furniture; manufacturing n.e.c.	Manufacturing
i37	Recycling of waste and scrap	Recycling	Manufacturing
i37.w.1	Recycling of bottles by direct reuse	Recycling	Manufacturing
i40.11.a	Production of electricity by coal	Electricity, gas, steam and hot water supply	Electricity and utilities
i40.11.b	Production of electricity by gas	Electricity, gas, steam and hot water supply	Electricity and utilities
i40.11.c	Production of electricity by nuclear	Electricity, gas, steam and hot water supply	Electricity and utilities
i40.11.d	Production of electricity by hydro	Electricity, gas, steam and hot water supply	Electricity and utilities
i40.11.e	Production of electricity by wind	Electricity, gas, steam and hot water supply	Electricity and utilities
i40.11.f	Production of electricity by petroleum and other oil derivatives	Electricity, gas, steam and hot water supply	Electricity and utilities

i40.11.g	Production of electricity by biomass and waste	Electricity, gas, steam and hot water supply	Electricity and utilities
i40.11.h	Production of electricity by solar photovoltaic	Electricity, gas, steam and hot water supply	Electricity and utilities
i40.11.i	Production of electricity by solar thermal	Electricity, gas, steam and hot water supply	Electricity and utilities
i40.11.j	Production of electricity by tide, wave, ocean	Electricity, gas, steam and hot water supply	Electricity and utilities
i40.11.k	Production of electricity by Geothermal	Electricity, gas, steam and hot water supply	Electricity and utilities
i40.11.l	Production of electricity nec	Electricity, gas, steam and hot water supply	Electricity and utilities
i40.12	Transmission of electricity	Electricity, gas, steam and hot water supply	Electricity and utilities
i40.13	Distribution and trade of electricity	Electricity, gas, steam and hot water supply	Electricity and utilities
i40.2	Manufacture of gas; distribution of gaseous fuels through mains	Electricity, gas, steam and hot water supply	Electricity and utilities
i40.3	Steam and hot water supply	Electricity, gas, steam and hot water supply	Electricity and utilities
i41	Collection, purification and distribution of water	Collection, purification and distribution of water	Services
i45	Construction	Construction	Construction
i45.w	Re-processing of secondary construction material into aggregates	Construction	Construction
i50.a	Sale, maintenance, repair of motor vehicles, motor vehicles parts, motorcycles, motor cycles parts and accessoiries	Sale, maintenance and repair of motor vehicles and motorcycles; retail sale services of automotive fuel	Transport
i50.b	Retail sale of automotive fuel	Sale, maintenance and repair of motor vehicles and motorcycles; retail sale services of automotive fuel	Transport
i51	Wholesale trade and commission trade, except of motor vehicles and motorcycles	Wholesale trade and commission trade, except of motor vehicles and motorcycles	Transport
i52	Retail trade, except of motor vehicles and motorcycles; repair of personal and household goods	Retail trade, except of motor vehicles and motorcycles; repair of personal and household goods	Transport
i55	Hotels and restaurants	Hotels and restaurants	Services
i60.1	Transport via railways	Land transport; transport via pipelines	Transport

i60.2	Other land transport	Land transport; transport via pipelines	Transport
i60.3	Transport via pipelines	Land transport; transport via pipelines	Transport
i61.1	Sea and coastal water transport	Water transport	Transport
i61.2	Inland water transport	Water transport	Transport
i62	Air transport	Air transport	Transport
i63	Supporting and auxiliary transport activities; activities of travel agencies	Supporting and auxiliary transport activities; activities of travel agencies	Services
i64	Post and telecommunications	Post and telecommunications	Services
i65	Financial intermediation, except insurance and pension funding	Financial intermediation, except insurance and pension funding	Services
i66	Insurance and pension funding, except compulsory social security	Insurance and pension funding, except compulsory social security	Services
i67	Activities auxiliary to financial intermediation	Activities auxiliary to financial intermediation	Services
i70	Real estate activities	Real estate activities	Services
i71	Renting of machinery and equipment without operator and of personal and household goods	Renting of machinery and equipment without operator and of personal and household goods	Services
i72	Computer and related activities	Computer and related activities	Services
i73	Research and development	Research and development	Services
i74	Other business activities	Other business activities	Services
i75	Public administration and defence; compulsory social security	Public administration and defence; compulsory social security	Services
i80	Education	Education	Services
i85	Health and social work	Health and social work	Services
i90.1.a	Incineration of waste: Food	Sewage and refuse disposal, sanitation and similar activities	Services
i90.1.b	Incineration of waste: Paper	Sewage and refuse disposal, sanitation and similar activities	Services
i90.1.c	Incineration of waste: Plastic	Sewage and refuse disposal, sanitation and similar activities	Services
i90.1.d	Incineration of waste: Metals and Inert materials	Sewage and refuse disposal, sanitation and similar activities	Services
i90.1.e	Incineration of waste: Textiles	Sewage and refuse disposal, sanitation and similar activities	Services
i90.1.f	Incineration of waste: Wood	Sewage and refuse disposal, sanitation and similar activities	Services

i90.1.g	Incineration of waste: Oil/Hazardous waste	Sewage and refuse disposal, sanitation and similar activities	Services
i90.2.a	Biogasification of food waste, incl. land application	Sewage and refuse disposal, sanitation and similar activities	Services
i90.2.b	Biogasification of paper, incl. land application	Sewage and refuse disposal, sanitation and similar activities	Services
i90.2.c	Biogasification of sewage slugde, incl. land application	Sewage and refuse disposal, sanitation and similar activities	Services
i90.3.a	Composting of food waste, incl. land application	Sewage and refuse disposal, sanitation and similar activities	Services
i90.3.b	Composting of paper and wood, incl. land application	Sewage and refuse disposal, sanitation and similar activities	Services
i90.4.a	Waste water treatment, food	Sewage and refuse disposal, sanitation and similar activities	Services
i90.4.b	Waste water treatment, other	Sewage and refuse disposal, sanitation and similar activities	Services
i90.5.a	Landfill of waste: Food	Sewage and refuse disposal, sanitation and similar activities	Services
i90.5.b	Landfill of waste: Paper	Sewage and refuse disposal, sanitation and similar activities	Services
i90.5.c	Landfill of waste: Plastic	Sewage and refuse disposal, sanitation and similar activities	Services
i90.5.d	Landfill of waste: Inert/metal/hazardous	Sewage and refuse disposal, sanitation and similar activities	Services
i90.5.e	Landfill of waste: Textiles	Sewage and refuse disposal, sanitation and similar activities	Services
i90.5.f	Landfill of waste: Wood	Sewage and refuse disposal, sanitation and similar activities	Services
i91	Activities of membership organisation n.e.c.	Activities of membership organisation n.e.c.	Services
i92	Recreational, cultural and sporting activities	Recreational, cultural and sporting activities	Services
i93	Other service activities	Other service activities	Services
i95	Private households with employed persons	Private households with employed persons	Services
i99	Extra-territorial organizations and bodies	Public administration and defence; compulsory social security	Services
